# SCeQTL: an R package for identifying eQTL from single-cell parallel sequencing data

**DOI:** 10.1186/s12859-020-3534-6

**Published:** 2020-05-11

**Authors:** Yue Hu, Xi Xi, Qian Yang, Xuegong Zhang

**Affiliations:** 1 MOE Key Laboratory of Bioinformatics and Bioinformatics Division, BNRIST, Department of Automation, Tsinghua University, Beijing, 100084 China; 2 School of Life Sciences, Center for Synthetic and Systems Biology, Tsinghua University, Beijing, 100084 China

**Keywords:** Single-cell eQTL, Zero-inflated negative binomial regression, Multi-class differential expression analysis, Single-cell gene regulation

## Abstract

**Background:**

With the rapid development of single-cell genomics, technologies for parallel sequencing of the transcriptome and genome in each single cell is being explored in several labs and is becoming available. This brings us the opportunity to uncover association between genotypes and gene expression phenotypes at single-cell level by eQTL analysis on single-cell data. New method is needed for such tasks due to special characteristics of single-cell sequencing data.

**Results:**

We developed an R package SCeQTL that uses zero-inflated negative binomial regression to do eQTL analysis on single-cell data. It can distinguish two type of gene-expression differences among different genotype groups. It can also be used for finding gene expression variations associated with other grouping factors like cell lineages or cell types.

**Conclusions:**

The SCeQTL method is capable for eQTL analysis on single-cell data as well as detecting associations of gene expression with other grouping factors. The R package of the method is available at https://github.com/XuegongLab/SCeQTL/.

## Background

Expression quantitative trait locus or eQTL analysis is an important approach for studying the association between variations in the genotype and gene expression, which may help to reveal the underlying regulation relationship. Technologies that can sequence in parallel both the genomes and transcriptomes of single cells are being developed recently [6, 8]. These technologies give us an opportunity to uncover the association between genetic variations and genes expression at single-cell level, which can help reveal detailed gene regulation mechanisms in processes like tumorigenesis and cell differentiation.

Methods for identifying eQTLs have been well studied for microarray data and bulk RNA-seq data. Typical methods of eQTL mapping include linear regression and ANOVA, where the expression level is taken as the dependent variable and the genotype at a single-nucleotide variation (SNV) site is the explaining factor [7, 16]. Most of those methods are based on the assumption that expression levels or its logarithms follow normal distribution, Poisson distribution or negative binomial distribution [17]. The Krux method used a non-parametric way to identify eQTL and claim their method is more robust [15]. These existing methods including the non-parametric ones can lose their power when applied on single-cell RNA-seq data because of the special characteristics of single-cell sequencing data, especially the excess of zero values.

The phenomenon of excess of zero values is common in single-cell RNA-seq (scRNA-seq) data [2, 9]. There are mainly two reasons. Because the amount of total RNAs in a single cell is extremely small, there is high probability that the RNA capture, reverse transcription and amplification steps may miss some transcripts, causing the expression of some expressed genes not observed in the sequencing data. This is usually called “drop-out” events. Another reason is that gene expression is a stochastic process at single-cell level [11]. This results in variations of gene transcription status between cells besides variations in gene expression abundances. The possibility for a gene to have a real zero expression level or to be in the “off” status of transcription is much higher in single cells than in the pooled transcriptomes of many cells in bulk RNA-seq data [5, 10]. There are two types of heterogeneity in gene expression: heterogeneity in the “on-off” status of a gene’s transcription, and heterogeneity in the abundance of expressed genes. Studying such heterogeneities is one of the major purposes of single-cell sequencing. Because of these special properties, when we analyze eQTLs on scRNA-seq data, we also face two possible types of differences in gene expression associated with variation in genotypes: differences in the transcription status of a gene and differences in expression levels of an expressed gene. We call them as “status difference” and “expression level difference”, respectively. We developed SCeQTL to analyze these two types of differences that may be associated with genotype variations. The method can also be applied to analyze associations of gene expression with other types of groupings such as cell lineages or cell types.

## Results

### Zero-inflated generalized linear model

We model scRNA-seq data as the outcome of two processes. One is that transcripts are captured in the sequencing and the corresponding gene gets non-negative expression values. The other is that transcripts are missed or the gene is not expressed in the cell, which will result in zero values in the data. The second process causes scRNA-seq data to have excess zero values. We find the negative binomial (NB) distribution can fit the non-zero parts of scRNA-seq data well in a way similar to bulk RNA-seq data (Fig. 3 and Fig. 4a), but there can be a high probability of a gene being zeros in the single-cell data.

Therefore, we use a zero-inflated negative binomial (ZINB) regression to model the scRNA-seq data as we have done in [10]. For gene expression *g* and genotype *s*, there is probability *p* that the transcription is off and we have the observation of a zero value in a cell, and probability 1 − *p* that the gene is expressed with values being described as following a negative binomial distribution. We call the probability *p* as zero ratio for simplicity. We write these as

$$ g\sim \left\{\begin{array}{c}0\  with\ probability\ p\\ {} NB\left(\mu, \theta \right)\  with\ probability\ 1-p\end{array}\right., $$

where *μ* and *θ* are the mean and shape parameter of the negative binomial distribution. We call a SNV to be an eQTL of a gene if the zero ratio *p* and/or the mean of negative binomial distribution *μ* is significantly correlated with the genotype of the SNV in a way that

$$ \ln \left(\frac{p}{1-p}\right)={\alpha}_1+{\beta}_1s $$

and/or

$$ \ln \left(\mu \right)={\alpha}_2+{\beta}_2s, $$

where *s* is the genotype (or other distinguishing factor to group the cells), parameters *α*_1_, *α*_2_, *β*_1_, *β*_2_ and the shape parameter *θ* are to be estimated from the data. Using maximum likelihood method to estimate the parameters, we get the log-likelihoods of the full model that includes the genotype as the explaining factor (*β*_1_ ≠ 0 or *β*_2_ ≠ 0) and of the reduced model that does not include the genotype (*β*_1_ = 0 *or β*_2_ = 0).

The generalized linear model contains two parts: distribution hypothesis and link function. In distribution hypothesis, the probability density function (pdf) of over-dispersed exponential families is

$$ p\left(x|\eta \right)=h\left(x,\sigma \right){e}^{\frac{\eta^TT(x)-A\left(\eta \right)}{\sigma }} $$

where *η* is a natural parameter, *T*(*x*) is a sufficient statistic, and *A*(*η*) is used to guarantee the integral of pdf to be 1. Link function describes how the expectation of corresponding variable is related to the linear combination of independent variables:

$$ \mathrm{E}\left(\mathrm{Y}\right)=\mu ={g}^{-1}\left( X\beta \right) $$

where *g* is the link function. For NB distribution, the link function is

$$ X\beta =\ln \left(\mu \right). $$

According to the generalized linear model theory [12], the deviance, which is − 2 times the log-likelihood ratio of the reduced model compared to the full model, follows an approximate chi-square distribution with *k* degree of freedom. The *k* is the difference between parameter numbers of the full model and the reduced model. We use the deviance as the test statistic to test for whether *β*_1_ = 0 or *β*_2_ = 0. By these two hypothesis tests, we can identify whether the gene expression have association with the genotype (or other factor used to group cells) and what kind of association it is. We call this method as SCeQTL and developed a software package in R to implement it (https://github.com/XuegongLab/SCeQTL/).

### Simulation experiments

Single-cell parallel sequencing technologies of both the genome and transcriptome are currently only available in very few labs, and the current genomic coverage of such technologies may not be sufficient for genotyping analysis yet. So it is still hard to find public datasets including both single-cell genomic sequencing data with sufficient depth and single-cell RNA-sequencing data of the same single cells. Therefore, we first did a series of simulation experiments to study the performance of SCeQTL. Both genotype and phenotype data were simulated simultaneously under different effect size. We applied SCeQTL and the widely used Matrix eQTL [16] on the simulation data for comparison. Matrix eQTL is a highly-efficient method for eQTL analysis designed for bulk data.

We simulated genotype and gene expression data of 1500 cells of three SNVs and 20 genes in each simulation experiment. Considering the probable effect of different frequencies of three genotypes (denoted by *s* = 0, 1, 2), we generated three SNVs (denoted as SNV 1–3) with different genotype frequencies, and conducted four experiments (denoted as Experiment 1–4) for each SNV. These experiments aimed to mimic four scenarios: transcription status set at same or different level while expression values change with genotypes, and expression values set at same or different level while transcription status change with genotypes. Under each scenario, we experimented on changes across different effect sizes. Ten significant gene-SNV pairs were randomly generated for each SNV, and were taken as the ground truth in performance analysis. Gene expression metrics were generated by ZINB model of different parameters. We define three types of genes that are associated with genotypes in the simulation model: genes whose expression values differ among genotypes (Ges), genes’ transcription status differs among genotypes (Gts), and both transcription status and expression values differ among genotypes (Gs). Table 1 shows the overall design of the simulation data.

**Table 1:** Simulation experiments description

SNVs	Experiments		Unchanged parameters	Changed parameters	Number of experiments
SNV 1 (freq. of s = 0/1/2: 0.25/0.5/ 0.25)	Experiment 1	Zero ratios set to the same (0.5). Differences in NB means among three genotypes increase.	*α*_1_ = 0	*β*_2_ ranges from 0 to 0.6, with increasing step of 0.05 across experiments.	13
			*β*_1_ = 0		
			*α*_2_ = 6		
	Experiment 2	Zero ratios set at different levels (0.5, 0.57 and 0.65). Differences in NB means among three genotypes increase.	*α*_1_ = 0		13
			*β*_1_ = 0.3		
			*α*_2_ = 6		
	Experiment 3	NB means set at the same level (403.43). Differences in zero ratios among three genotypes increase.	*α*_2_ = 6	*β*_1_ ranges from 0 to 0.6, with increasing step of 0.05 across experiments.	13
			*β*_2_ = 0		
			*α*_1_ = 0		
	Experiment 4	NB means set at different levels (403.43, 601.85 and 897.85). Differences in zero ratios among three genotypes increase.	*α*_2_ = 6		13
			*β*_2_ = 0.4		
			*α*_1_ = 0		
SNV 2 (0.16/0.48/0.36)	Same as in SNV 1				
SNV 3 (0.09/0.42/0.49)	Same as in SNV 1				

**Fig. 1:**
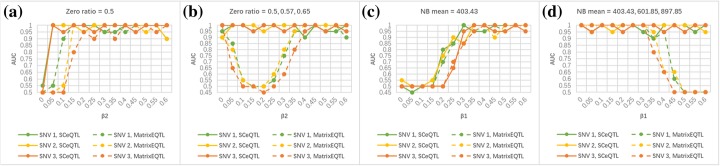
AUC values of Experiment 1–4 for SNV 1–3. **a** AUC values of Experiment 1 for SNV 1–3. **b** AUC values of Experiment 2 for SNV 1–3. **c** AUC values of Experiment 3 for SNV 1–3. **d** AUC values of Experiment 4 for SNV 1–3

For each experiment, we get the ROC curves of both methods by calculating the false positive rate and true positive rate by comparing the detection result with the simulation model. We use Area Under Curve (AUC) of ROC curves to demonstrate performance of the two methods. Larger AUC value means higher accuracy. Figure 1 summarizes the experiment results. It shows that different proportions of genotypes do not affect results. Further checking the results in four experiments of the same SNV, we can see that when only one aspect (zero ratio or NB mean) differs (Fig. 1a, c), performance of the two methods largely overlaps: AUC value first rises, then holds with effect sizes increasing. A minor difference shown in Fig. 1a is that the power of SCeQTL rises earlier and more dramatically at smaller effect sizes. This indicates higher sensitivity of SCeQTL than Matrix eQTL for detecting Ges.

In more complicated situations when both transcription status and expression values are different (Fig. 1b, d), AUC value of SCeQTL keeps steady and high, while that of Matrix eQTL drops drastically at certain effect sizes. This suggests the superiority of SCeQTL in terms of power when detecting Gs. An explanation is that, in Fig. 1b, the increase of zero ratio gradually offset the divergence of NB mean in three genotypes with *β*_2_ increasing; and in Fig. 1d, the increase of NB mean gradually offset the divergence of zero ratio with *β*_1_ increasing. Both cases resulted in similar mean in three genotypes which Matrix eQTL cannot distinguish. But once the influence of zero ratio or NB mean became dominant, the power of Matrix eQTL would recover, as is shown in the right part of curves in Fig. 1b and the left part of curves in Fig. 1d.

**Fig. 2:**
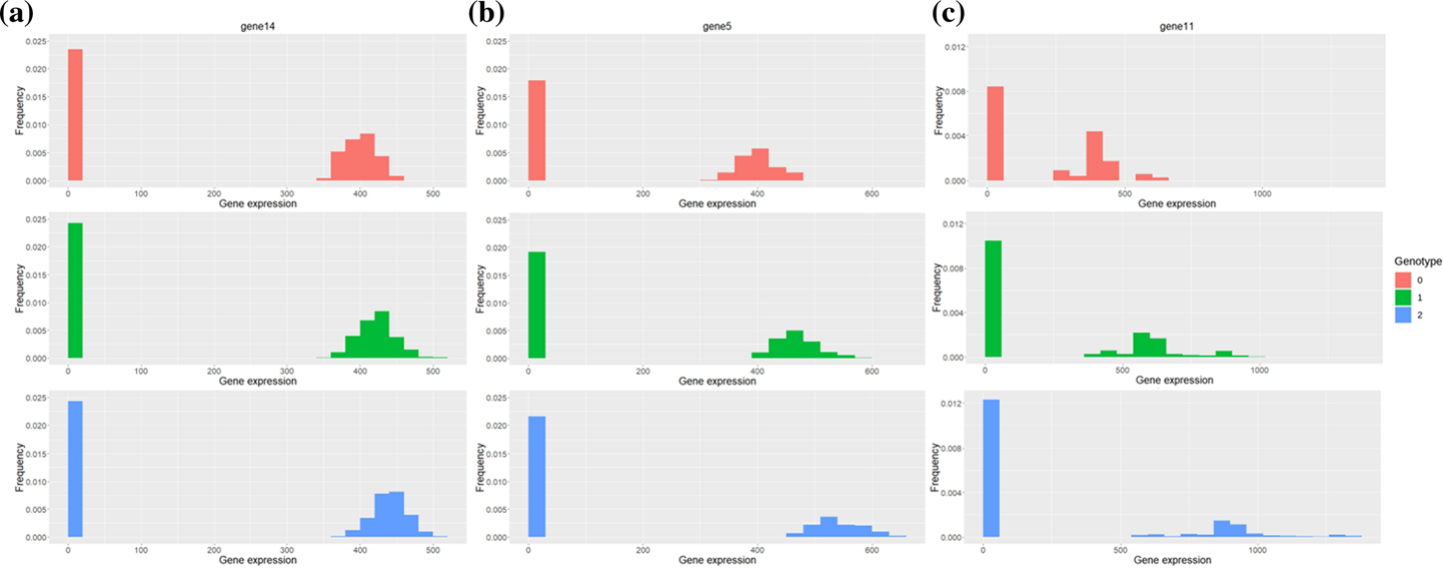
Examples of Ges and Gs for SNV 2 where SCeQTL found significant, but Matrix eQTL did not. **a** Ge (gene14) in Experiment 1, corresponding to the point in Figure 1a when *β*_2_ = 0.05. Only expression levels are different. **b** G (gene5) in Experiment 2, corresponding to the point in Figure 1b when *β*_2_ = 0.15. Both transcription status and expression levels are different. **(c)** G (gene11) in Experiment 4, corresponding to the point in Figure 1d when *β*_1_ = 0.5. Both transcription status and expression levels are different

Figure 2 shows gene expression distributions of three example significant eQTLs which cannot be detected by Matrix eQTL. Figure 2a shows expression levels of a Ge referred to the point in Fig. 1a when *β*_2_ = 0.05. SCeQTL can detect the slight difference in NB mean, but Matrix eQTL cannot. Figure 2b and c display similar situations when detecting Gs. They correspond to the point in Fig. 1b when *β*_2_ = 0.15 and Fig. 1d when *β*_1_ = 0.5, respectively. Again, SCeQTL found the differences very significant, while Matrix eQTL found them insignificant. They could support our analyses above.

### Real data experiments

Currently public datasets with both genotype and transcriptome sequenced in the same single cells are still rare, and those only few available datasets still have very limited coverage in SNVs. We therefore used a real RNA-seq dataset without cell-level genotype but with multiple groups of cell attributes to further study the performance of SCeQTL. The data we used is a scRNA-seq dataset of human preimplantation embryo cells of different embryo days [14]. We split the cells into three groups according to the embryonic day (E5, E6 and E7) to mimic cells of three genotypes. We use this dataset to show that SCeQTL not only works for single-cell eQTL applications, but also can be applied to the more general scenarios of detecting gene expression variations that are associated with other types of grouping factors of cells.

**Fig. 3:**
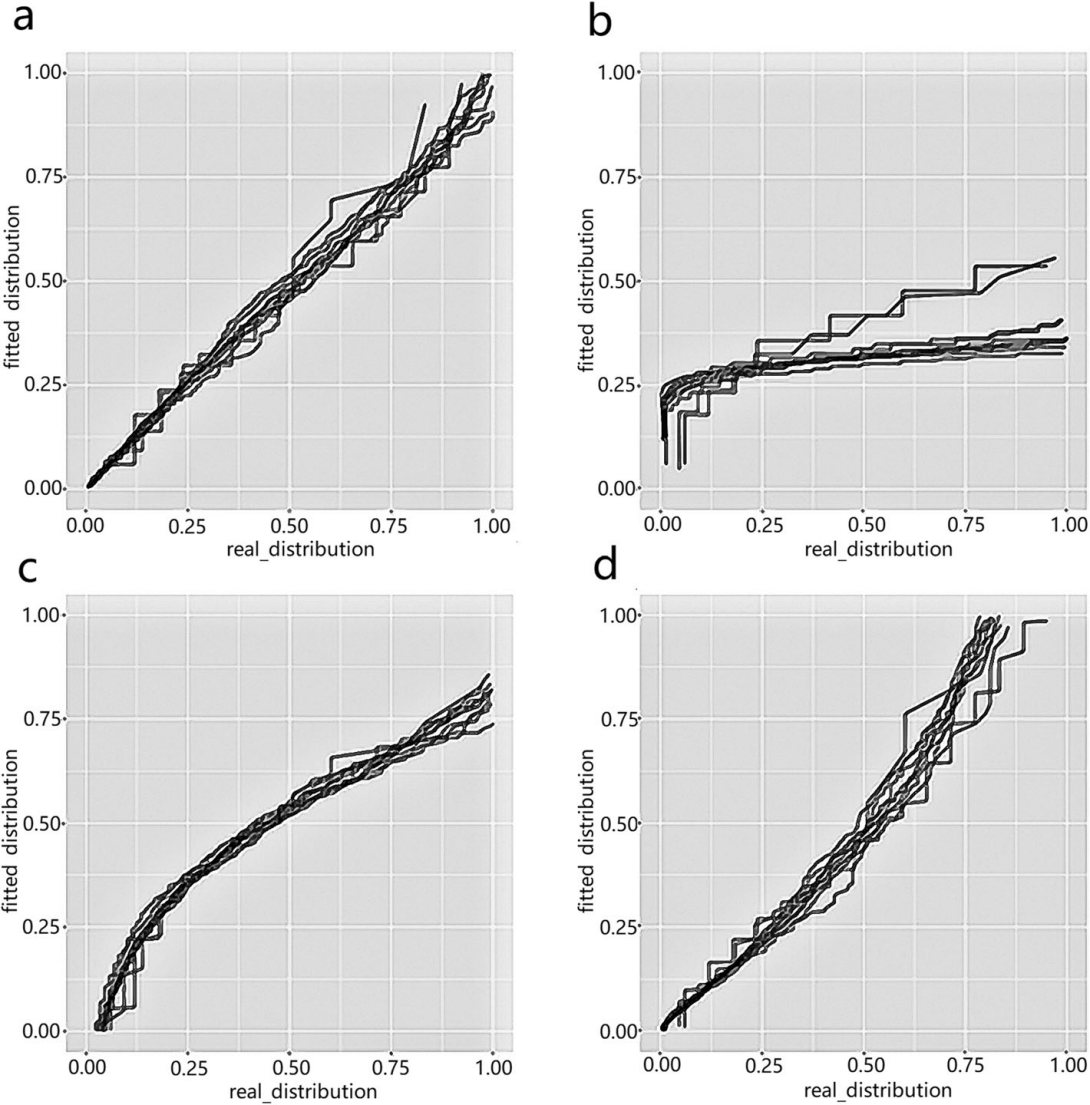
Q-Q plot of scRNA-seq data with (**a**) Negative Binomial distribution (**b**) Poisson distribution (**c**) Normal distribution (**d**) Log-normal distribution

**Fig. 4:**
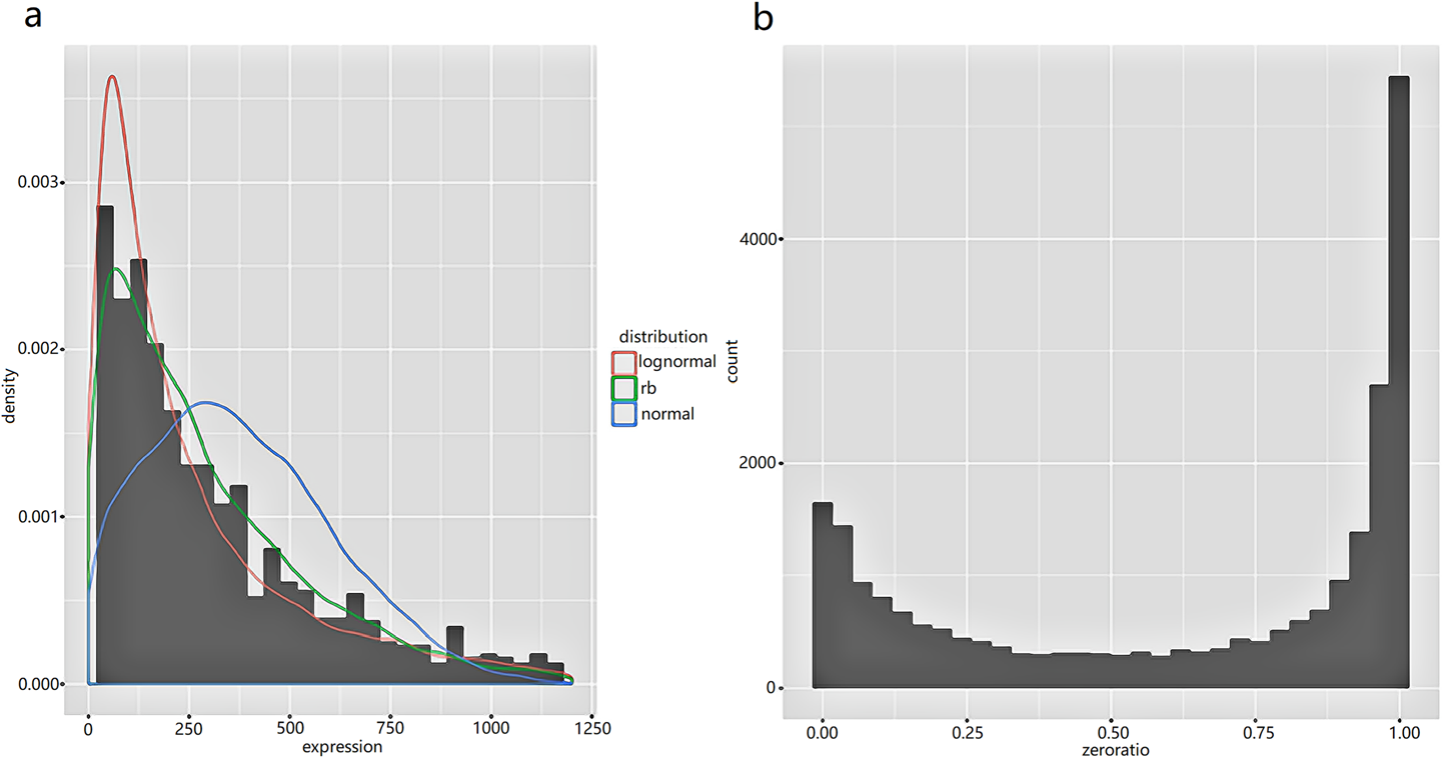
**a** Probability density function of fitted distributions and the histogram of a sample; **b** Histogram of the proportion of zeros of all the genes

We first checked whether the non-zero expression data were fitted well with our model using the ‘checkdist’ function in our package. We randomly picked some genes and drew Q-Q plot to compare gene expression distribution with several distributions. Figure 3 and Figure 4a shows that negative binomial distribution is appropriate for modeling the non-zero part of the data, while the lines in Q-Q plot of other distributions are far away from the diagonal. The histogram in Figure 4b shows that the drop-out event is very common in single-cell RNA-seq data and needs to be considered.

**Fig. 5:**
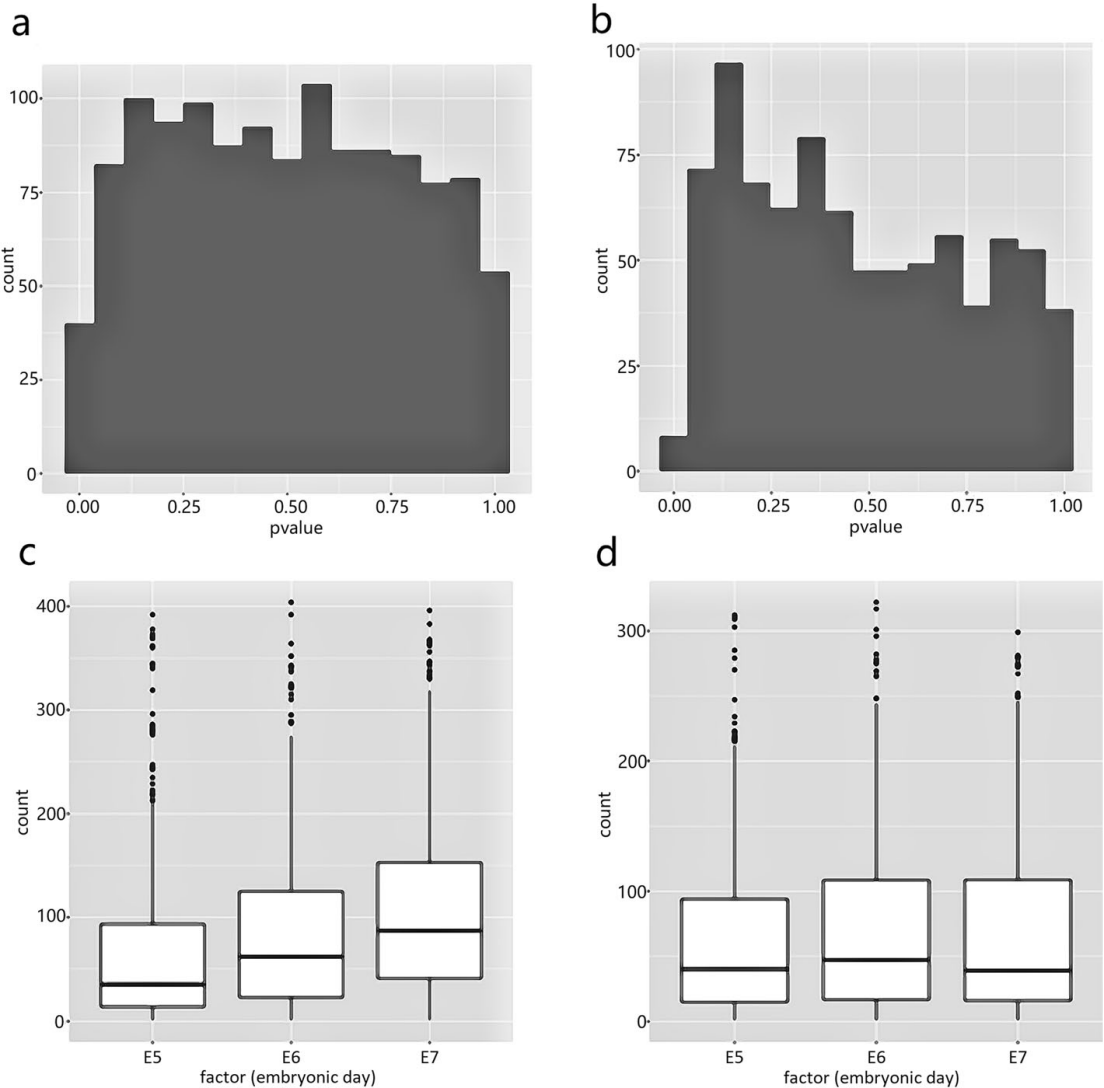
*P*-value distribution under null hypothesis of (**a**) SCeQTL and (**b**) Matrix eQTL. **c** An example that non-zero part of gene expression have significant differences among genotype groups. **d** The non-zero part of an example of significant zero-ratio differences. Zero-ratio of three genotype groups are 0.76, 0.26 and 0.26, respectively. We can see from the figure that the non-zero expressions are not associated with the genotype, but the zero-ratios are. These findings will be not significant for Matrix eQTL after multiple test correction, but are very significant for SCeQTL

We applied both SCeQTL and Matrix eQTL [16] on these data for comparison. We first conducted experiments to study the distribution of *p*-values of the two methods under the null hypothesis of no eQTL. Figure 5 show the *p*-value distributions under null hypothesis, which were obtained by randomly generating and permuting the “genotype” (the embryonic days in this experiment) and use two methods to calculate the *p*-values. The *p*-value distribution of SCeQTL is close to uniform distribution between 0 and 1, while the *p*-value distribution of Matrix eQTL has clear deviation from uniform.

On the eQTL results in the experiment of true embryonic day with gene expression, we found that results of the two methods largely overlapped, but there were noticeable cases on which SCeQTL worked better. Figure 5c shows an example that non-zero part had significant difference but Matrix eQTL didn’t find it. The *p*-values obtained by SCeQTL and Matrix eQTL are 9.37 × 10^−9^ and 0.002, respectively. The test has been done for all the 23,981 genes in this dataset. This eQTL is very significant for SCeQTL but will not significant for Matrix eQTL after multiple-test correction. One reason is that the negative binomial distribution fit the single-cell data better than normal distribution. On the other hand, the zero values in scRNA-seq data caused the means of the three groups to be almost equal, so that Matrix eQTL could not detect the difference. Figure 5d gives an example that zero ratios have significant differences among the compared groups (0.76, 0.26 and 0.26) but non-zero parts shown by the boxplots are almost same. The *p*-values with SCeQTL and Matrix eQTL are 1.09 × 10^−45^ and 0.004, respectively. Matrix eQTL can’t detect differences of this type either.

**Fig. 6:**
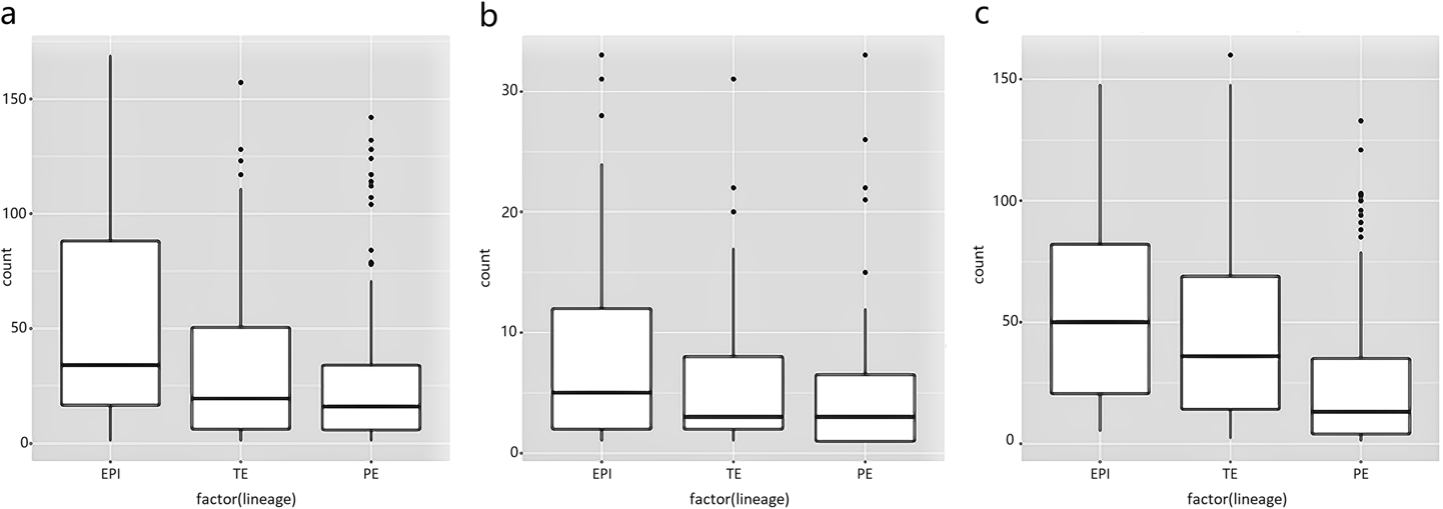
Examples of lineage-associated genes identified by SCeQTL. **a** PSORS1C2: Zero-ratio of three lineages are 0.18, 0.85, and 0.57, respectively. **b** PTPRZ1: Zero-ratio of three genotype groups are 0.45, 0.93 and 0.73, respectively. **c** AMDHD1: Zero-ratio of three genotype groups are 0.60, 0.79, and 0.38, respectively

We also experimented on this dataset using cell lineage as the factor to distinguish three cell groups, and found SCeQTL could successfully find results that can be confirmed with biological knowledge on embryonic cell lineages. By dividing the samples by cell lineages EPI (epiblast), TE (trophectoderm) and PE (primitive endoderm), we applied SCeQTL to find genes that vary among different cell lineages. Among all 23,981 genes, SCeQTL found about 20 genes with *p*-value less than 10^−40^, 70 genes with *p*-value less than 10^−30^ and 200 genes with *p*-value less than 10^−20^. In these genes that are significantly associated with cell lineages, we found some have been reported as lineage specific genes in the literature. For example, for EPI-specific genes PRDM14, GDF3, TDGF1, NODAL, SOX2 and NANOG, their *p*-values are 5.7 × 10^−45^ 、 4.7 × 10^−20^, 6.5 × 10^−16^, 4.0 × 10^−21^, 5.1 × 10^−37^ and 4.8 × 10^−25^; for TE-specific genes GATA2, GATA3 and DAB2, their *p*-values are 4.1 × 10^−20^, 6.7 × 10^−26^ and 3.3 × 10^−21^; and for PE-specific genes HNF1B, PDGFRA and GATA4, their *p*-values are 5.7 × 10^−32^, 1.7 × 10^−23^ and 2.0 × 10^−35^, respectively. All these lineage-specific genes ranked at top 200 in our result. It is worth noting that quite a lot of these genes have shown obvious transcription status differences in our SCeQTL analysis, which may imply that gene transcription in single cells are undergone on-off regulation in many scenarios. To double check the reliability of the SCeQTL discoveries, we manually checked some of the results and found most significant genes truly have differences among the compared groups. Figure 6 shows three examples. *P*-values of SCeQTL in the examples are 4.1 × 10^−50^, 4.8 × 10^−33^ and 1.5 × 10^−23^ respectively. These experiments showed the potential for discovering associations in single cells that cannot be identified using existing eQTL methods. The biological implication of those associations can be important and deserve further investigations.

## Discussion

A limitation of the proposed SCeQTL method is that the computation cost is relatively high if applied for eQTL analysis at whole-genome scale. It can take a few minutes to analyze a few hundred gene-SNV pairs on a single computing node. This is mainly due to the iterative procedures in estimating the parameters. However, for most single-cell studies, the cells are from the same tissue sample or closely related samples. We can expect that the number of SNVs among the cells that need to be studied for eQTL analysis is not too large to make the computing cost of SCeQTL a severe issue in practical applications. This will also not be an issue when we use SCeQTL to analyze the association of gene expression with other factors as we did in the application examples.

The analysis of the associations of genotypic variations with gene expression as well as alternative splicing [18] is a fundamental step for understanding the complex gene regulation system of human in health and disease. Cells are the basic units where the regulation happens. The broad existence of heterogeneities in gene expression both in quantity and in alternative splicing isoforms among cells is important in human physiology and pathology. This gives a strong motivation why functional genomics studies are moving quickly into single-cell levels. This is also true for the study of gene regulations. The current single-cell genomics technologies are still at their early stages and not widely available for large-scale studies of gene regulations within single cells, but technologies are developing and evolving rapid toward these goals. We hope the proposed SCeQTL method and software provides a ready and effective tool for this development.

## Conclusions

We proposed a new method for eQTL analysis on single-cell genomic and transcriptomic parallel sequencing data and developed a software package SCeQTL to implement the method. Experiments showed that the method can reveal associations that cannot be identified with existing eQTL methods developed for bulk data. It can also be applied on tasks of finding the association of gene expression with other grouping factors that distinguish cells into different types. It provides an effective tool for exploring gene regulation relationships at single-cell level.

## Methods

### Data preprocessing

Multiple steps of data pre-processing are necessary before using SCeQTL. Firstly, we remove the effect of the library size. We use the normalization method in DEseq [1] for this normalization. The median of the ratios of observed counts is used to measure the sequencing depth.

$$ {s}_j= media{n}_i\frac{g_{ij}}{{\left({\prod}_{v=1}^m{g}_{iv}\right)}^{\frac{1}{m}}} $$

where *g*_*ij*_ is the expression level of gene i in sample j. The denominator is obtained by calculating the geometric mean across non-zero samples. As discussed in [1], this method is more robust than just taking the sum of all genes as sequencing depth since otherwise the highly expressed gene would dominant the result, which is often seen in single-cell gene expression data. All samples are normalized by the size factor, and we round down the resulted expression values to fit our read-counts model.

Next, single-cell RNA-seq data are noisy and we need to remove genes and variants which are not suitable for the analysis. Genes with read counts less than a certain threshold (by default, <=1) are treated as not expressed and are therefore removed. We only consider genes whose variances are greater than a certain threshold (by default, > = 5). For genomic variants, only variants with at least two genotypic groups in the dataset and each genotype has at least 5 samples (cells) are further considered.

When we enter the iteration of analyzing every gene-variant pairs, pairs that don’t have enough non-zero values (by default, <=5) in one genotype are reported. The estimation of distribution parameters can be far away from true values in this situation. And we find that in real data, there are samples whose expression level is much higher than the others. If we include these samples into consideration, the mean of negative binomial distribution will be overestimated. So we treat these samples as outlier and use robust z score as defined below to remove them (by default, > = 4), where MAD stands for the median absolute deviation:

$$ {z}_{robust}=\frac{g_{ij}- media{n}_j\left({g}_{ij}\right)}{MA{D}_j\left({g}_{ij}\right)} $$

### Parameter estimation

The package ‘pscl’ [19] is used to estimate the parameter and calculate the log-likelihood. The package uses the EM algorithm or BFGS algorithm to iteratively update the parameter estimation.

### Covariates correction

It is common that some hidden covariates may exist in the sampled population, such as age, gender, or other clinical variables. It is important to remove the effect of them from the eQTL study, as otherwise a high percentage of results will be false discoveries. SCeQTL allows user to define a covariate vector x as possible confounding factors to be considered in the analysis. With covariate vector x ∈ R^n^, the models become

$$ \ln \left(\frac{\mathrm{p}}{1-\mathrm{p}}\right)={\upalpha}_1+{\upbeta}_1\mathrm{s}+{\sum}_{\mathrm{i}=1}^{\mathrm{n}}{\upgamma}_{1\mathrm{i}}{\mathrm{x}}_{\mathrm{i}}, $$

$$ \ln \left(\upmu \right)={\upalpha}_2+{\upbeta}_2\mathrm{s}+{\sum}_{\mathrm{i}=1}^{\mathrm{n}}{\upgamma}_{2\mathrm{i}}{\mathrm{x}}_{\mathrm{i}}, $$

where extra parameter vectors ***γ***_**1**_ and ***γ***_**2**_ to be estimated. The hypothesis test process is the same as non-covariates one. As a special consideration in single-cell studies, potential correlations among single cells from the same individual or from the same cell type can be modeled in this covariate vector to make sure that the associations detected with SCeQTL are not due to those factors.

### Multiple test correction

We provide two ways to control the false discovery, Benjamini-Hochberg (BH) method [3] and the q-value method. The q-value method is implemented by R package ‘qvalue’ (http://github.com/jdstorey/qvalue). Since several publications come up with other methods for multiple test correction in eQTL mapping [4, 13], users can also select whether to let SCeQTL to report *p*-value or false discovery rates and set the threshold according to other correction methods.

## Data Availability

The R package of the method is available at https://github.com/XuegongLab/SCeQTL/.

## Abbreviations

SCeQTL: Single Cell expression Quantitative Trait Locus
scRNA-seq: Single cell RNA sequencing
ANOVA: ANalysis Of VAriance
MAD: Median absolute deviation
EM: Expectation-Maximization
BFGS: Broyden–Fletcher–Goldfarb–Shanno
